# Selective CNS Uptake of the GCP-II Inhibitor 2-PMPA following Intranasal Administration

**DOI:** 10.1371/journal.pone.0131861

**Published:** 2015-07-07

**Authors:** Rana Rais, Krystyna Wozniak, Ying Wu, Minae Niwa, Marigo Stathis, Jesse Alt, Marc Giroux, Akira Sawa, Camilo Rojas, Barbara S. Slusher

**Affiliations:** 1 Brain Science Institute, Johns Hopkins School of Medicine, Baltimore, Maryland, United States of America; 2 Department of Neurology, Johns Hopkins School of Medicine, Baltimore, Maryland, United States of America; 3 Department of Molecular and Comparative Pathobiology, Johns Hopkins School of Medicine, Baltimore, Maryland, United States of America; 4 Department of Psychiatry and Behavioral Sciences, Johns Hopkins School of Medicine, Baltimore, Maryland, United States of America; 5 Department of Neuroscience, Johns Hopkins School of Medicine, Baltimore, Maryland, United States of America; 6 Kurve Technology, Inc., Bothell, Washington, United States of America; University of Pécs Medical School, HUNGARY

## Abstract

Glutamate carboxypeptidase II (GCP-II) is a brain metallopeptidase that hydrolyzes the abundant neuropeptide N-acetyl-aspartyl-glutamate (NAAG) to NAA and glutamate. Small molecule GCP-II inhibitors increase brain NAAG, which activates mGluR3, decreases glutamate, and provide therapeutic utility in a variety of preclinical models of neurodegenerative diseases wherein excess glutamate is presumed pathogenic. Unfortunately no GCP-II inhibitor has advanced clinically, largely due to their highly polar nature resulting in insufficient oral bioavailability and limited brain penetration. Herein we report a non-invasive route for delivery of GCP-II inhibitors to the brain via intranasal (i.n.) administration. Three structurally distinct classes of GCP-II inhibitors were evaluated including DCMC (urea-based), 2-MPPA (thiol-based) and 2-PMPA (phosphonate-based). While all showed some brain penetration following i.n. administration, 2-PMPA exhibited the highest levels and was chosen for further evaluation. Compared to intraperitoneal (i.p.) administration, equivalent doses of i.n. administered 2-PMPA resulted in similar plasma exposures (AUC_0-t, i.n_./AUC_0-t, i.p._ = 1.0) but dramatically enhanced brain exposures in the olfactory bulb (AUC_0-t, i.n_./AUC_0-t, i.p._ = 67), cortex (AUC_0-t, i.n_./AUC_0-t, i.p._ = 46) and cerebellum (AUC_0-t, i.n_./AUC_0-t, i.p._ = 6.3). Following i.n. administration, the brain tissue to plasma ratio based on AUC_0-t_ in the olfactory bulb, cortex, and cerebellum were 1.49, 0.71 and 0.10, respectively, compared to an i.p. brain tissue to plasma ratio of less than 0.02 in all areas. Furthermore, i.n. administration of 2-PMPA resulted in complete inhibition of brain GCP-II enzymatic activity *ex-vivo* confirming target engagement. Lastly, because the rodent nasal system is not similar to humans, we evaluated i.n. 2-PMPA also in a non-human primate. We report that i.n. 2-PMPA provides selective brain delivery with micromolar concentrations. These studies support intranasal delivery of 2-PMPA to deliver therapeutic concentrations in the brain and may facilitate its clinical development.

## Introduction

Elevated levels of glutamate, a major neurotransmitter in the central and peripheral nervous system, is often associated with excitotoxicity, which is a hallmark of many neurological and psychiatric disorders [1–3]**.** One strategy to reduce the levels of extracellular glutamate involves the inhibition of the brain enzyme glutamate carboxypeptidase II (GCP-II) (EC 3.4.12.21), a membrane bound zinc metalloprotease involved in the hydrolysis of the abundant neuropeptide N-acetylaspartylglutamate (NAAG) to N-acetylaspartate (NAA) and L-glutamate [1,4,5]. NAAG is released from neurons/axons after depolarization [6] and acts as an agonist at presynaptic metabotropic glutamate 3 receptors (mGluR3) [7] which limits further glutamate release, although controversy exists around this finding [8,9]. Released NAAG can also be catabolized by GCP-II, liberating glutamate, which can serve as an agonist at various glutamate receptors. Inhibition of GCP-II results in both increased extracellular NAAG and decreased extracellular glutamate. Both of these effects dampen glutamate transmission and can afford neuroprotection. In support of this, small molecule inhibitors of GCP-II have been demonstrated to be efficacious in multiple preclinical models wherein excess glutamate transmission is implicated including traumatic spinal cord and brain injury [10–12] stroke [4], neuropathic and inflammatory pain [13–27], ALS [28], schizophrenia [29], neuropathy [30,31], drug abuse [32–35] and cognition [36]. In addition, GCP-II knockout animals have shown to be protected against ischemic brain injury, peripheral neuropathy [37], and have demonstrated long term memory enhancing effects [38].

Several GCP-II inhibitors with different chemical scaffolds have been synthesized over the last two decades including those with phosphonate (e.g. 2-(phosphonomethyl)-pentanedioic acid, 2-PMPA), thiol (e.g. 2-(3-mercaptopropyl)pentane-dioic acid; 2-MPPA) and urea moieties (e.g. (N-[N-[(S)-1,3-dicarboxypropyl]carbamoyl]-L-cysteine; DCMC) [5]. Potent GCP-II inhibitors identified to date have required two functionalities–a glutarate moiety that binds the C-terminal glutamate recognition site of GCP-II, and a zinc chelating group to engage the divalent zinc atoms at the enzyme’s active site [5]. Although inclusion of these functionalities has led to highly potent inhibitors, the compounds suffer from being exceedingly hydrophilic and show low membrane permeability. The only GCP-II inhibitor class to show oral bioavailability was the thiol-based inhibitors, with 2-MPPA advancing into clinical studies [39]. Unfortunately, subsequent immunological toxicities (common to thiol drugs) were observed in primate studies which halted its development. The phosphonate based inhibitor 2-PMPA is extremely potent (IC_50_ = 300 pM.), selective [4,13], and has demonstrated therapeutic benefit in over twenty *in vivo* models of neurological disorders performed by several independent laboratories[4,15–17,40–44]. Despite its picomolar potency, most preclinical studies have administered 2-PMPA at doses of 50–100 mg/kg i.p. or i.v. to produce therapeutic effects, as the compound is highly hydrophilic and has limited oral bioavailability and tissue penetration [45]. Similar limitations have been met with urea-based inhibitors, which have mainly been utilized as peripheral imaging agents [46].

The pressing need to move these efficacious, but hydrophilic compounds into the clinic, led us to search for alternative patient compliant routes of administration. Intranasal delivery to the brain is non-invasive and offers several advantages including avoidance of hepatic first pass clearance, rapid onset of action, frequent self-administration and easy dose adjustments [47]. Intranasal administration of a number of small molecules, macromolecules, gene vectors and cells has been shown to be successful in animal and clinical studies [48–54]. Small molecules have an added advantage of being absorbed paracellularly through the nasal epithelium after which, these molecules can then directly enter the CNS through the olfactory or the trigeminal nerve associated pathway [47]. Small molecules like Lidocaine, Losartan, Deferoxamine, and Remoxipride have shown to be directly transported to the brain upon intranasal administration [54–57]. Employing a similar strategy, we first compared the brain penetration of three potent and widely used GCP-II inhibitors using intranasal delivery in a single dose and single time-point study. 2-PMPA exhibited the highest levels in the preliminary analysis and was chosen for more detailed characterization. We report that compared to i.p. administration, equivalent i.n. administered dose of 2-PMPA resulted in similar plasma but dramatically enhanced brain exposures. These findings were then confirmed in non-human primate studies.

## Materials and Methods

2-PMPA, 2-PMSA (internal standard), and 2-MPPA and were synthesized internally by our laboratory as reported previously [13,58]. DCMC was donated by Dr. Martin Pomper at Johns Hopkins University. Losartan (internal standard) was obtained from Sigma-Aldrich (St. Louis, MO). LC/MS grade acetonitrile and water (LC/MS grade) with 0.1% formic acid were obtained from Fisher Scientific (Hanover Park, IL). Drug-free (blank) heparinized rat plasma was obtained from Innovative Research Inc. (Plymouth, MN). All other chemical and reagents were purchased from Sigma-Aldrich (St. Louis, MO).

### Animal studies

All of the animal studies in rodents were performed as per protocols approved by the Institutional Animal Care and Use Committee (Protocol# RA13; **S1 Protocol**) at Johns Hopkins University and primate studies were conducted in accordance with the guidelines recommended in *Guide for the Care and Use of Laboratory Animals* (National Academy Press, Washington DC, 2011) following approval by Animal Care and Use Committee (Protocol # 031637) at Ricerca Biosciences (Concord, OH, USA).

#### Rodent i.n. and i.p. dosing

Studies were conducted in male Wistar rats (6–8 weeks; weighing 200–250 g) obtained from Harlan Laboratories (Indianapolis, IN) that were maintained in a controlled environment and allowed food and water ad libitum. Intranasal administrations were performed according to previously described methods with minor modifications [59,60]. Briefly, rats were anesthetized with a 1–1.5 mL intraperitoneal (i.p.) dose of 10% chloral hydrate (approved under the protocol RA#13), and kept under anesthesia with additional chloral hydrate as needed, throughout the entire experiment. To prevent drainage of nasally dosed solution, the nasal cavity was isolated from the respiratory and gastrointestinal tracts. An incision was made along the neck, and the trachea isolated and transected. The upper part was tied off with a 3–0 silk suture, and the lower part cannulated with PE240 tubing to aid air breathing. Rats were maintained lying on their back, and their heads were maintained in supine position, and in this position, given 10 μL (375 mg/mL) of the experimental drug solution per nostril using a micro-syringe connected to 1.5 cm PE-10 tube, over a period of 5–10 s. The rats were maintained under anaesthesia for the entire duration of the experiment until sacrificed. The total dose received was 30 mg/kg for each drug solution.

For i.p. studies 2-PMPA was administered as a single i.p. dose. All dosing solutions were prepared on the day of the experiment in 50 mM HEPES buffered saline, and pH adjusted to 7.4 before injection. At various time points following drug administration (0.16, .5, 1, 3, 5 h) post dose, animals (n = 3 per time point, except 3 h n = 2 animals) were euthanized with CO_2_, and blood samples were collected in heparinized microtubes by cardiac puncture and tissues (olfactory bulb, frontal cortex, and cerebellum) were dissected and immediately flash frozen (-80°C). Plasma was prepared by centrifugation immediately after collection of blood samples. All samples were stored in -80°C until bioanalysis.

#### Rodent ex vivo GCP-II enzymatic activity

One half of the brain tissues collected following i.n. administration (1h post dose) was used to determine GCP-II NAAG hydrolyzing activity. In brief, tissues were weighed and immersed in 0.5 mL of ice-cold 50 mM Tris Buffer (pH 7.7 at RT). Each tissue was sonicated for 30–60 seconds using an ultrasonic cell disrupter. After a 2 minute spin at 13,000 g, supernatants were analyzed for protein content and NAAG-hydrolyzing activity measurements were performed as previously described [61,62].

#### Non-human i.n. primate dosing

The study was conducted in accordance with the guidelines recommended in *Guide for the Care and Use of Laboratory Animals* (National Academy Press, Washington DC, 2011). Briefly, (**S1 ARRIVE Checklist**) A male cynomolgus monkey (approximately 3.5 kg, non-drug naive) was housed in a stainless steel cage (size 30” wide x 31” deep x 31.5” high) maintaining temperature of 64–84°F, humidity of 30–70% with alternating 12-hour light/dark cycle as per the USDA Animal Welfare Act (9 CFR, Parts 1, 2, and 3). Food was provided twice daily in amounts appropriate for the size and age of the animals and tap water was available *ad libitum*. To provide psychological enrichment, monkey was provided television entertainment for at least 1 hour per day, (at least 2 to 3 times weekly); received fruits, vegetables, and additional treats minimally 3 times weekly; and housed with rubber toys on a full-time basis throughout the duration of the study. The health status of the animal was evaluated in accordance with accepted veterinary practice; no abnormalities were observed throughout the study. Following the last sample collection, the animal was released to the facility stock animal colony. The animal was healthy and was not sacrificed. The study was conducted by Michael Stonerook, Ph.D., D.V.M., DABT, the technical director at Ricera Biosciences, LLC.

The monkey was sedated with 45 mg of ketamine and 0.25 mg of midazolam given as an intramuscular injection prior to test article administration. Sedation was maintained through blood and cerebrospinal fluid (CSF) sample collections with ketamine/midazolam at a starting rate of 20 mg/kg/h ketamine and 0.4 mg/kg/h midazolam. 2-PMPA was administered as an aqueous solution (500 mg/mL and pH adjusted to 7.4) via i.n. delivery employing a drug delivery device (Kurve Technology, Bothell, Washington), designed to deliver drugs to the olfactory region to maximize transport to the central nervous system [63]. The device was actuated for a period of 2 min in one nostril (depositing 100 μL). The nose piece was cleaned with a mist of air and then the same procedure was performed in the second nostril (100 μL). Total dose delivered was 100 mg. CSF sample (target of 50 μL) was obtained by an indwelling cannula placed in the intrathecal space at the cisterna magna at 0.5 h post dose. Blood was collected via venipuncture of the femoral vein at 0.5 h post dose and plasma was obtained by low speed centrifugation at 1500 x g for 10 minutes. The plasma was flash-frozen on dry ice after separation. Plasma and CSF samples were stored in a freezer set at -70°C, until bioanalysis.

### Bioanalysis of DCMC, 2-MPPA, and 2-PMPA in rodent plasma and brain

For quantification of analytes in plasma and brain tissues, extraction was performed using protein precipitation and subsequently processed for analysis by LC/MS/MS. Briefly, prior to extraction, frozen samples were thawed on ice. For plasma samples, 50 μL of the calibration standard or sample were transferred into silanized vials. For brain tissues, the samples were weighed in a 1.7 mL silanized vials to which 4 times the volume of methanol (dilution 1:5) was added. The tissues were stored in -20°C for 1 h and then homogenized. The calibration curves were developed using plasma and brain from untreated animals as a matrix. For plasma, sample preparation involved a single liquid extraction by addition of 300 μL of methanol as extraction solution with internal standard, followed by vortexing for 30 s and then centrifugation at 12000 x g for 10 min. Supernatant was transferred and evaporated to dryness at 40°C under a gentle stream of nitrogen. For brain tissue, homogenized samples were vortexed and centrifuged as above, and 100 μL supernatant was mixed with 100μL of internal standard in methanol, and then evaporated to dryness at 40°C under a gentle stream of nitrogen. For 2-PMPA analysis, samples were derivatized to improve sensitivity and enable reverse phase chromatography **S1 Fig**. For derivatization, the residue was reconstituted with 100 μL of n-butanol with 3N HCl and samples were vortexed. The samples were heated at 60°C in a shaking water bath for 30 min. At the end of 30 min the derivatized samples were dried under a gentle stream of nitrogen. 2-MPPA and DCMC were processed without additional derivatisation step and were amenable to reverse phase chromatography. Following extraction of 2-MPPA, DCMC, and derivatized 2-PMPA, the residue was reconstituted in 100 μL of 30% acetonitrile and water v/v. The samples were vortexed and centrifuged. Supernatant (75μL) was transferred to a 250 μL polypropylene autosampler vial sealed with a Teflon cap and a volume of 10 μL was injected onto the ultra-performance liquid chromatography (UPLC) instrument for quantitative analysis using a temperature-controlled autosampler operating at 10°C.

Chromatographic analysis was performed using an Accelaultra high-performance system consisting of an analytical pump, and an autosampler coupled with TSQ Vantage mass spectrometer (Thermo Fisher Scientific Inc., Waltham MA). Separation of the analyte was achieved at ambient temperature using Agilent Eclipse Plus UPLC column (100 x 2.1mm i.d.) packed with a 1.8 μm C18 stationary phase. The mobile phase was composed of 0.1% formic acid in acetonitrile and 0.1% formic acid in H_2_O with gradient elution. The total run time for each analyte was 5.0 min. The [M+H]^+^ ion transitions of derivatized 2-PMPA (m/z 339.537>191.354, 149.308), and the internal standard (m/z 325.522 >121.296,195.345); DCMC (m/z 309.416 >119.272,130.321) and the internal standard (335.460 > 145.28, 188.32) and the [M-H]^-^ion transitions for 2-MPPA, at (m/z 205.300 >171.459, 187.462) and the internal standard (m/z 421.670 >127.368, 179.576) were monitored by LC/MS/MS. Calibration curves over the range of 0.034–17.0 μg/mL for DCMC; and 0.021–10.3 μg/mL for 2-MPPA in brain tissue; and 0.011–22.6 μg/mL for 2-PMPA in plasma and tissue were constructed from the peak area ratio of the analyte to the internal standard using linear regression with a weighting factor of 1/(nominal concentration). Correlation coefficient of greater than 0.99 was obtained in all analytical runs for all analytes. The mean predicted relative standard deviation for back calculated concentrations of the standards for all analytes were within the range of 85 to 115%, except for the lowest concentration which was within the range of 80 to 120%.

### Pharmacokinetic analysis of 2-PMPA in rodents

Mean plasma and tissue concentrations of 2-PMPA were analyzed using non-compartmental method [64] as implemented in the computer software program WinNonlin Professional version 5.0.1 (Pharsight Corp., Mountain View, CA). The maximum plasma concentration (C_max_) and time to C_max_ (T_max_) were the observed values. The area under the plasma concentration time curve (AUC) value was calculated to the last quantifiable sample (AUC_last_) by use of the log-linear trapezoidal rule. The brain to plasma partition coefficients were calculated as a ratio of their AUCs (AUC_0-t,brain_/AUC_0-t,plasma_). The elimination half-life (t_1/2_) was determined by dividing 0.693 by λ _z_.

### Analysis of 2-PMPA in primate plasma and CSF

For analysis of 2-PMPA in CSF samples no derivatization was performed as CSF offers a much cleaner matrix compared to brain and plasma which are complex. Standard curves were generated in blank artificial CSF. Calibration curves were constructed in the range of 0.113–22.6 μg/mL. Samples were analysed on agilent QTOF mass spectrometer by LC/MS. Samples (20 μL) were injected and separated on an Agilent 1290 LC equipped with an Agilent Eclipse Plus C18 column (2.1 X 100 mm) packed with 1.8 micron stationary phase. The mobile phase consisted of 0.1% formic acid in water and 0.1% formic acid in acetonitrile with an isocratic elution at 2.5% organic. Analytes were detected with an Agilent 6520 QTOF mass spectrometer in negative mode using [M-H]^-^ ions for 2-PMPA (225.0163) and the internal standard (325.1043). Calibration curves were generated with a correlation coefficient >0.99 in a similar manner as described above. Plasma analysis was conducted in a similar manner as described above (rodent plasma 2-PMPA analysis; **S1 Fig**) using naïve male cynomolgus monkey plasma for standard curve.

## Results

### Brain concentrations of i.n. administered DCMC, 2-MPPA and 2-PMPA in rodents

**Fig 1:**
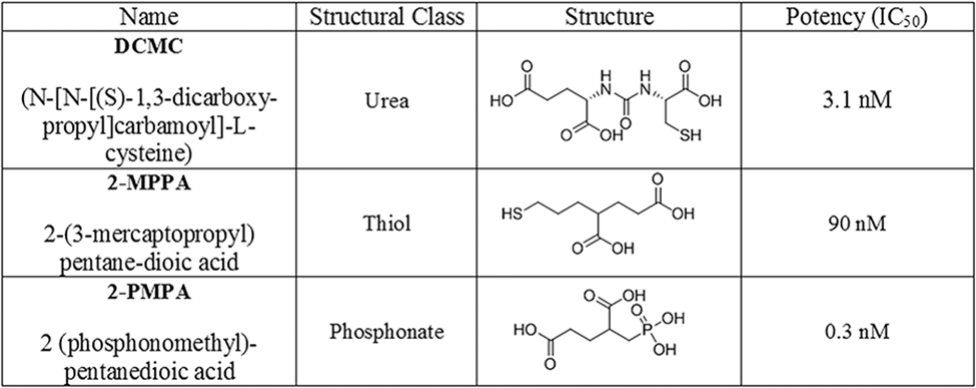
Chemical structures and IC_50_ values of DCMC, 2-MPPA, 2-PMPA.

**Fig 2:**
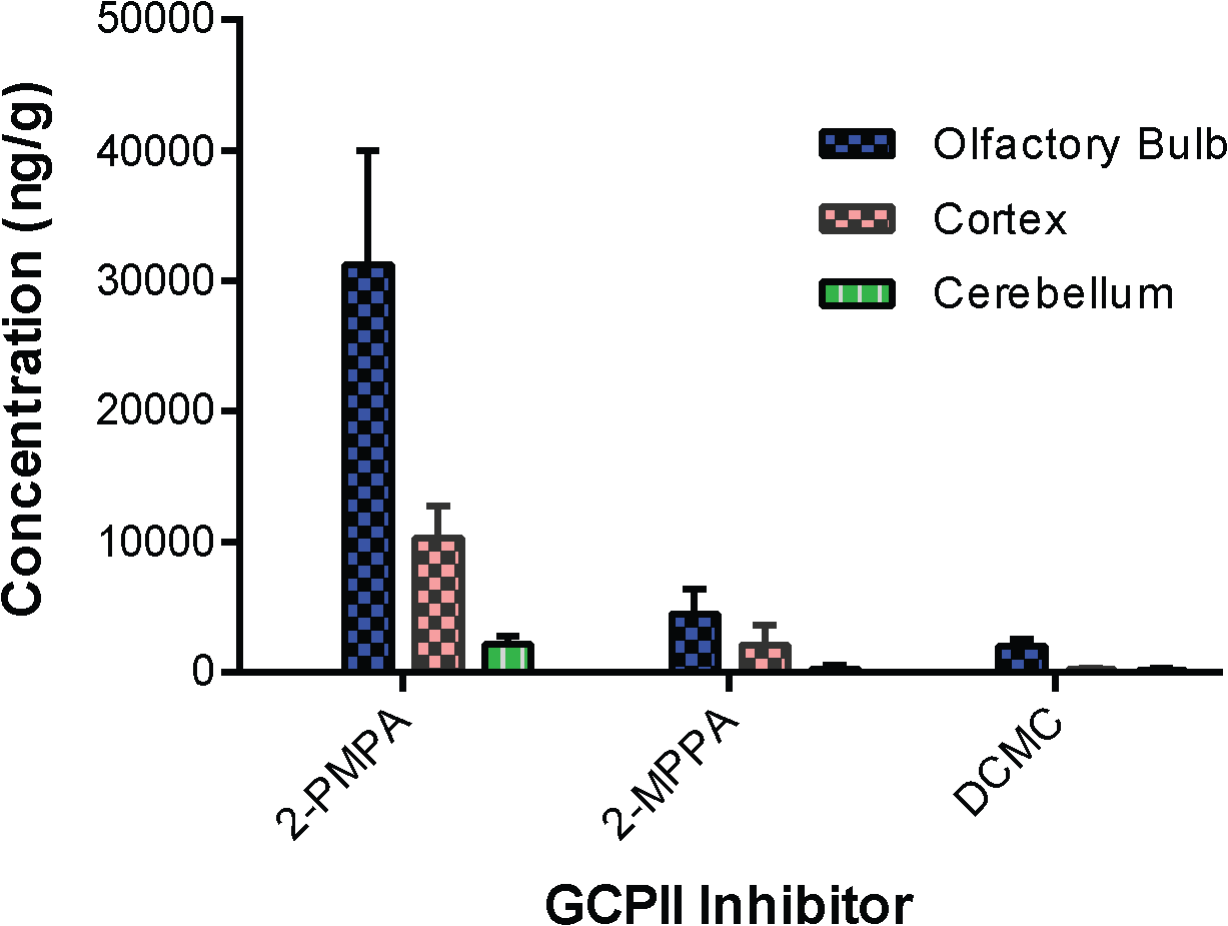
Mean concentrations of 2-PMPA, 2-MPPA and DCMC in different brain regions. Concentration were measured in olfactory bulb, cortex and cerebellum following 30mg/kg intranasal administration in rats. Tissues were collected 1h post dose and evaluated via LC/MS/MS.

The structures of three chemically distinct GCP-II inhibitors DCMC, 2-MPPA and 2-PMPA and their IC_50_ values are shown in **Fig 1**. DCMC, 2-MPPA and 2-PMPA were evaluated in a single time point (1 h post dose) pharmacokinetic study in rats dosed i.n. at 30 mg/kg. While all three inhibitors showed some brain penetration, 2-PMPA exhibited the highest levels (**Fig 2)**. As shown in **Fig 2**, at 1 h following i.n. administration, 2-PMPA was found in the olfactory bulb, cortex and cerebellum at 31.2 μg/g, 10.3 μg/g and 2.13 μg/g, respectively. 2-MPPA and DCMC showed less exposure with 4.46 μg/g and 2.12 μg/g, 0.26 μg/g and 2.03 μg/g, and 0.21 μg/g and 0.20 μg/g in the olfactory bulb, cortex and cerebellum, respectively.

### Comparison of the pharmacokinetics of i.p. versus i.n. administered 2-PMPA in rodents

**Fig 3:**
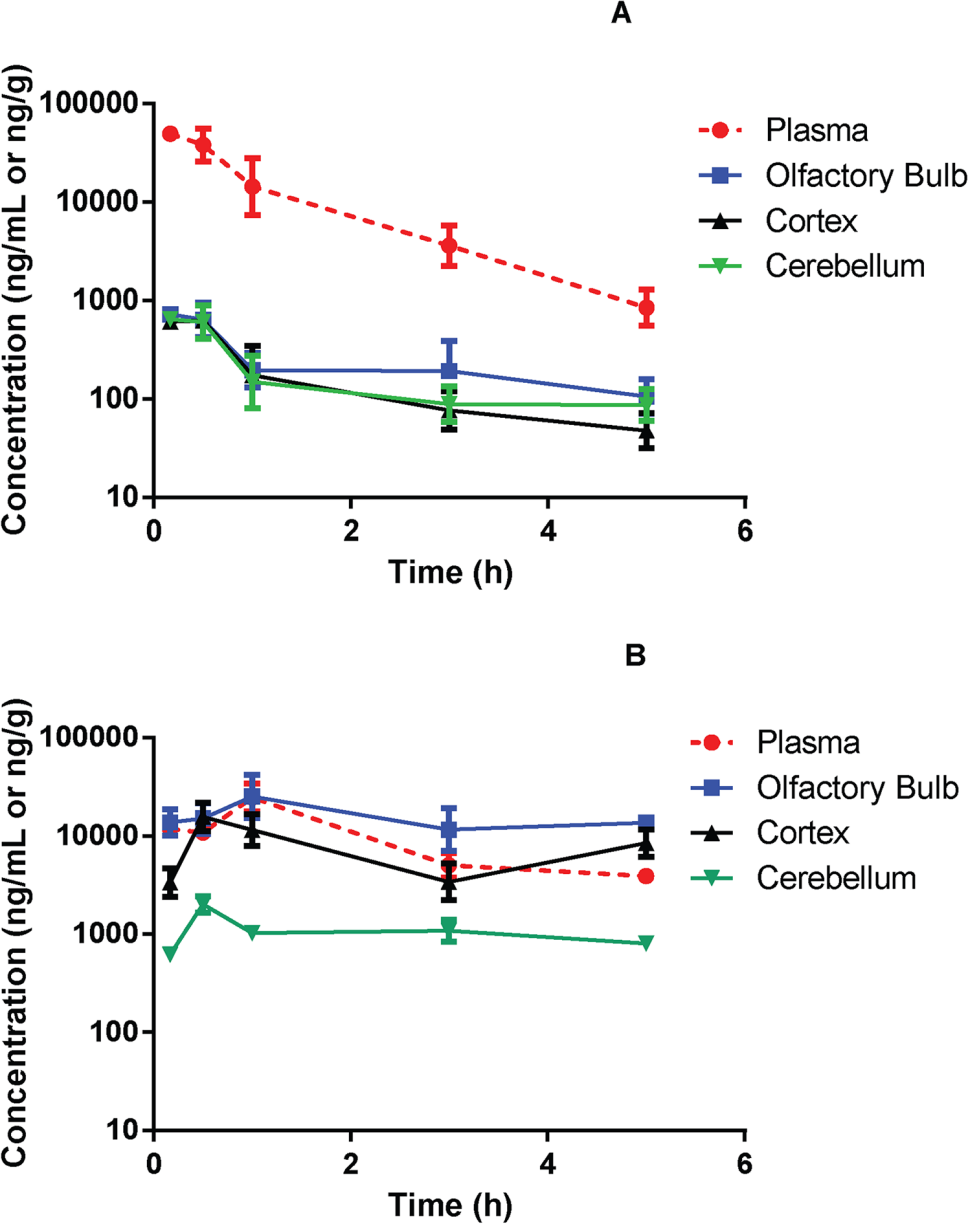
Mean concentration vs. time profiles for 2-PMPA in rat plasma, olfactory bulb, cortex and cerebellum following (A) 30 mg/kg intraperitoneal (i.p.) and (B) 30 mg/kg intranasal (i.n.) administration.

**Fig 4:**
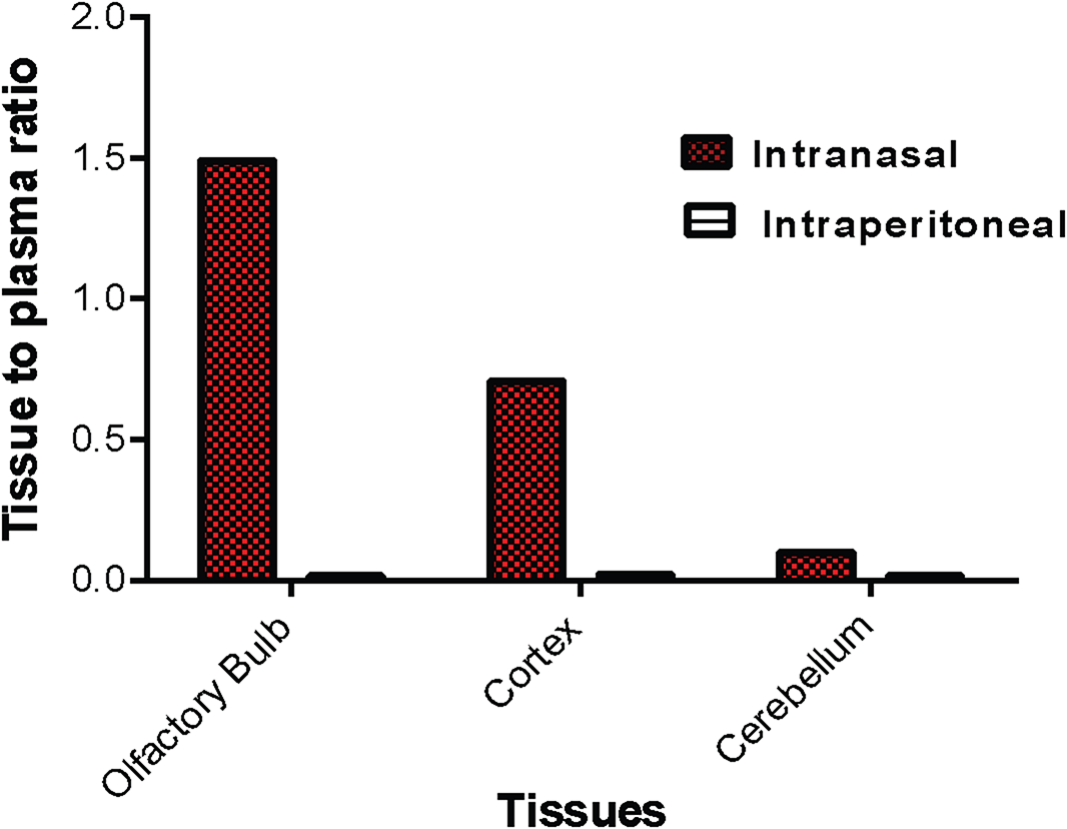
Brain tissue to plasma (B/P) ratio of 2-PMPA in different brain regions. B/P ratio was calculated based on area under the curve (AUC_**0-t**_) following 30 mg/kg i.n. or i.p. administration.

Pharmacokinetic studies of 30 mg/kg 2-PMPA in rat plasma and brain tissues following i.n. and i.p. administration were conducted and compared. Similar to what we have previously demonstrated [45], i.p. administered 2-PMPA showed rapid absorption in plasma with peak plasma concentration (C_max_) of 49.5 μg/mL observed at the first time point of 0.167 h. The AUC_0-t_ achieved for plasma was 50.3 h*μg/mL and the elimination t_1/2_ value was 0.99 h depicting rapid elimination. The apparent volume of distribution was low (0.82 L/kg) and the apparent clearance was rapid (9.71 mL/min/kg). The AUC_0-t_ achieved for olfactory bulb, cortex and cerebellum were 1.15 h*μg/g, 0.84 h*μg/g, and 0.80 h*μg/g respectively (**Fig 3A**). The brain tissue to plasma ratios based on AUCs (AUC_0-t,brain_/AUC_0-t,plasma_) was less than 0.02 for olfactory bulb, cortex, and cerebellum (**Fig 4)**.

Following i.n. administration, the 2-PMPA plasma C_max_ was 24.7 μg/mL observed at 1 h post dose. The plasma AUC_0-t_ was 52.3 h*μg/mL. The AUC_0-t_ for olfactory bulb, cortex and cerebellum were 78.1 h*μg/g, 37.7 h*μg/g and 5.27 h*μg/g respectively (**Fig 3B)**. The brain tissue to plasma ratios based on AUCs (AUC_0-t,brain_/AUC_0-t,plasma_) were 1.49, 0.71 and 0.10 in the olfactory bulb, cortex, and cerebellum respectively (**Fig 4)**. The elimination t_1/2_ value and apparent clearance were not reported due to the lack of elimination phase following intranasal route.

### GCP-II functional activity in rodent brain following 2-PMPA i.n. administration

**Fig 5:**
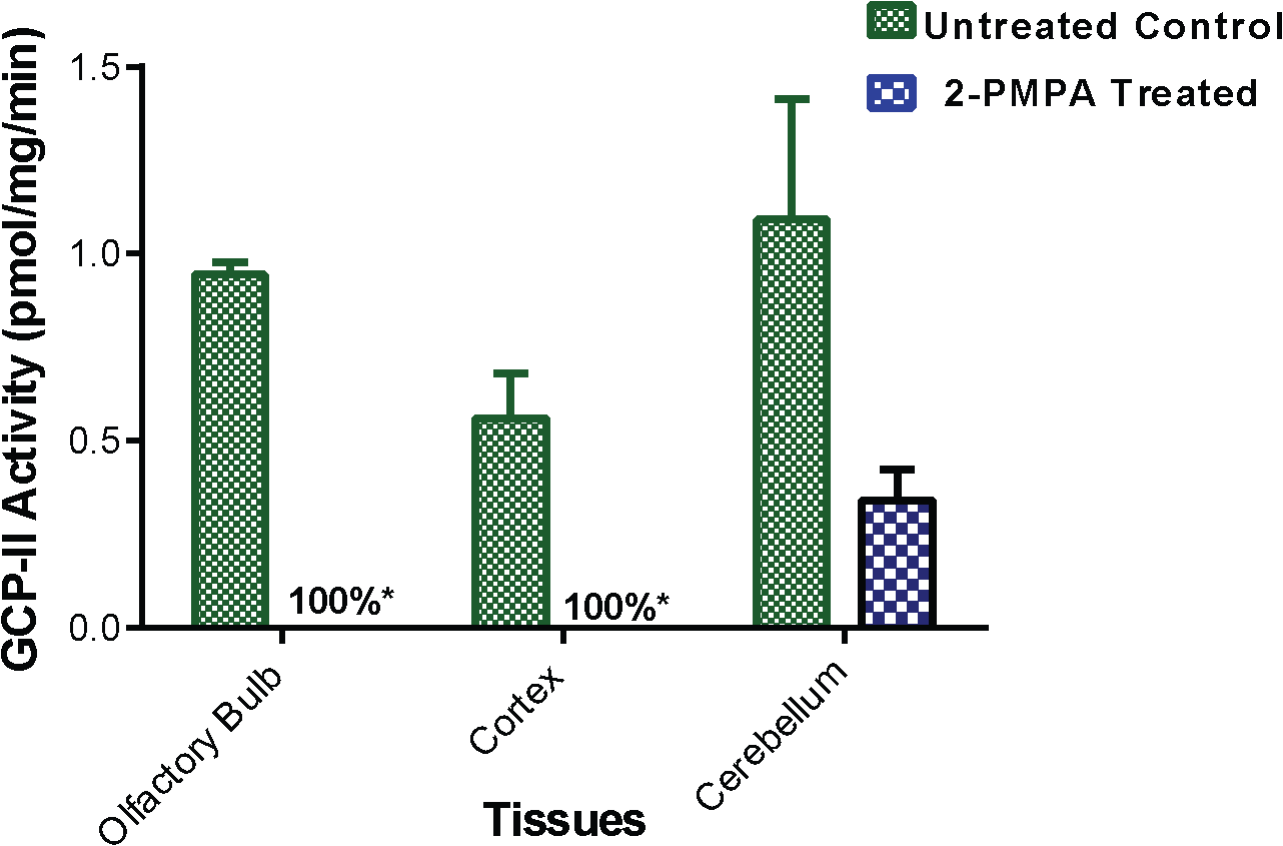
Ex vivo GCP-II enzymatic activity following 2-PMPA i.n. administration. Enzyme activity was measured in olfactory bulb, cortex and cerebellum collected 1 h post dose following 30 mg/kg i.n. administration. Percent inhibition was calculated in all tissue samples relative to brain tissues collected from untreated control rats.

Target engagement studies were performed by measurement of GCP-II enzymatic activity in brain tissue 1 h following i.n. 2-PMPA administration (**Fig 5)**. There was complete (100%) inhibition of GCP-II activity measured in olfactory bulb and cortex following i.n. administration and almost complete (70% ± 5%) inhibition in the cerebellum.

### CSF exposure of 2-PMPA following i.n. administration in non-human primates

In an exploratory study conducted by Ricerca Biosciences, LLC, 2-PMPA was administered to a male cynomolgus monkey using the Vianase™ intranasal device at a total dose of 100 mg. Following i.n. administration at 30 min post dose, the plasma level of 2-PMPA was below the limit of quantitation (<50 nM), while the CSF concentration was 0.32 μg/mL (~1.5μM) determined by LC/MS/MS.

## Discussion

GCP-II (also termed NAALADase or NAAG peptidase) is a 94kD class II membrane bound zinc metalloenzyme that modulates glutamatergic transmission through its NAAG hydrolyzing activity in the CNS [5]. Inhibition of GCP-II has shown to provide neuroprotection both by increasing brain NAAG and modulating mGluR3 receptor activity, and by decreasing glutamate release [4]. Potent small-molecule GCP-II inhibitors have demonstrated therapeutic utility in over twenty preclinical models of neurological disorders demonstrated independently by several laboratories [5]. Unfortunately, the rational design of GCP-II inhibitors with glutarate and zinc chelating moieties has resulted in poor physicochemical properties, including extreme hydrophilic nature, with limited oral bioavailability and blood–brain barrier (BBB) penetration. Rigorous efforts led to the design of thiol based inhibitors which were found to be orally bioavailable in preclinical species [65,66], of which 2-MPPA was evaluated in clinical studies [39]. Unfortunately, its development was halted due to membranoproliferative glomerular nephritis, thought to be immune complex mediated, observed in non-human primates. As a class, thiol drugs have a known risk of inducing immunotoxicity and hypersensitivity reactions [67,68]. Thus, in spite of finding numerous potent and efficacious molecules, to date no GCP-II inhibitor has advanced into clinical studies.

To overcome these challenges and to aide in the transition of our potent, selective, and efficacious small molecule GCP-II inhibitors into the clinical setting, we have examined alternative patient compliant routes for CNS delivery. One such alternative non-invasive mechanism is the nasal route for delivery of drugs to the brain via the olfactory region, since the olfactory receptor cells are in direct contact with both the nasal environment and the central nervous system (CNS) [48]. As a result, delivery of biologics, peptides, and small molecules from the nasal passages to the brain have now been documented in numerous animal and clinical studies [49,52,57,63].

We first assessed intranasal drug delivery of three structurally distinct classes of GCP-II inhibitors including a urea, thiol, and phosphonate based inhibitor namely DCMC, 2-MPPA and 2-PMPA, respectively. All three compounds share common glutarate functionality but with a different zinc chelating group (Fig 1). Of the three compounds delivered intranasally, 2-PMPA showed the highest penetration in the brain tissues followed by 2-MPPA and DCMC. Given the similarity in the polar nature of the compounds, 2-PMPA’s preferential brain uptake intranasally was not obvious. Based on the current understanding of the transport of hydrophilic molecules delivered intranasally [49,52,60,69], 2-PMPA is most likely transported via the extracellular pathways along the peripheral olfactory or trigeminal nerves. While this is an extracellular pathway, it may also present barriers to penetrability that can create differences in efficiencies between similar molecules, but is not as restrictive as BBB [52,70]. It is important to note, that most drugs given by intranasal route have quite low bioavailability based on dose administered, with the overall quantity appearing in the brain tissue normally less than 1% [70,71,72] of the administered dose. It is perhaps not surprising that small differences in physiochemical properties may result in large differences in delivery efficiency as was observed here.

Using racemic 2-PMPA, we then conducted a time course evaluation following intranasal administration and directly compared it to a systemic i.p. route. 2-PMPA has been evaluated in several preclinical models using i.p. route of administration and has generally shown efficacy at 50–100 mg/kg [5] despite its picomolar potency *in vitro*. This could be explained in part due to low brain to plasma ratio of 2-PMPA of ≤ 2% following systemic administration [45]. Our results illustrate significant differences in the pharmacokinetics of 2-PMPA following i.n. versus i.p. administration. As seen in Fig 3A and 3B, both plasma and brain tissues had detectable concentration within the first 10 min. Following i.p. route there was almost 2 orders of magnitude difference in the concentrations measured in plasma vs brain tissue suggesting low extent of partitioning into brain via systemic route similar to our previous findings [45]. When directly comparing the i.p. vs i.n. route, based on AUC_0-t_, the plasma exposures from the two routes were same. However a dramatic difference was observed in brain penetration. Most importantly, the nasal route led not only to an increase in absolute exposures (increased total brain concentration compared to i.p. route) but also in relative exposures (increased brain to plasma partition ratio compared to i.p. route). Further, by pooling the data from plasma and brain following i.p. and i.n. route, it is apparent that most of the 2-PMPA reaching the brain was from direct i.n. route through the olfactory pathway and only 2% of it is accounted from the plasma. Another notable difference following i.n. administration was the slower elimination compared to the i.p. route perhaps due to the existence of a slow continuing absorption process from the nasal mucosa. This profile indicates absorption rate-limited elimination (flip-flop kinetics) as has been previously described via i.n. route [54]. These results are promising and provide a clinical path forward for an extremely hydrophilic and a potent compound like 2-PMPA, that has been highly efficacious in many preclinical models of neurological disorders, but whose translation into clinic has been hampered due to poor physiochemical properties.

Most studies investigating the pathway from the nose to the brain have been performed in rodents. However in comparison to the human nose, rodent nose offers a significantly higher surface area to volume ratio and a significantly higher percentage of nasal epithelium devoted to olfaction. Furthermore, in humans, the olfactory region is located in the roof of the nasal cavity while the olfactory area in rats is spread throughout the posterior part of the cavity. These anatomical differences are important and should be taken into consideration for correct interpretation of results from rodent models [52]. One model species that has been recently used and is anatomically similar to humans is non-human primates. We performed a pilot primate study using a device specifically designed for targeting the olfactory region that is currently employed in clinical studies [63]. This device, known as Vianase developed by Kurve Inc., is the liquid drug delivery system based on Controlled Particle Dispersion technology. Our exploratory study in a non-human primate using the olfactory targeting device revealed selective permeation with 1.5 μM concentrations in primate CSF and undetectable levels in plasma (<50 nM) at 30 min post dose. These intital data, although limited, are promising representing selective delivery to CSF with >100 fold concentration versus the IC_50_ for 2-PMPA. CSF is quite often used as a surrogate for brain concentrations; however, due to complexity in intranasal pathways, predictions of correlative brain concentrations are not feasible with this data. A drug given intranasally, can take multiple pathways to the brain including direct, through olfactory and/or trigeminal nerve, or can pass indirectly through respiratory epithelium to the plasma and then through BBB to the brain. Drugs can traverse from olfactory to CSF or brain to CSF and vice versa. Depending on the properties of drug molecules one or more pathways are likely to dominate. Thus in order to elucidate the mechanism of 2-PMPA delivery via intranasal route to the CSF and brain, and to get quantitative the measurements in primate brain radiolabeled agents such as radiofluorinated 2-PMPA [73] may provide a more viable option. Future studies to evaluate such mechanisms are underway.

Overall our i.n. studies suggest a direct pathway for the transfer of 2-PMPA via the olfactory mucosa into the CNS. Intranasal drug-delivery provides a promising strategy to achieve therapeutic 2-PMPA concentrations in the brain and may facilitate its development in clinic. In addition to its therapeutic potential, intranasal administration of 2-PMPA may also have diagnostic potential as an imaging agent in the brain when attached to a suitable isotope for GCPII localization, or characterization of its changes in neurological disease.

## Supporting Information

S1 ARRIVE Checklist NCR3s The ARRIVE Guidelines Checklist. (PDF)

S1 Fig Derivatization process for 2-PMPA bioanalysis. Reaction was carried out using n-butanol with 3N HCl at 60°C for 30 min, leading to formation of n-butyl esters of 2-PMPA carboxylic acids.
(TIF)

S1 Protocol Approved rat protocol (RA13M65). Pharmacokinetics and behavioral characterization of proprictary small molecule therapeutics in rats.
(PDF)
