# Supplementary figures and images for: Selective CNS Uptake of the GCP-II Inhibitor 2-PMPA following Intranasal Administration
Source: PLoS One. 2015 Jul 7;10(7):e0131861. doi: 10.1371/journal.pone.0131861 (PMC4494705; doi:10.1371/journal.pone.0131861)

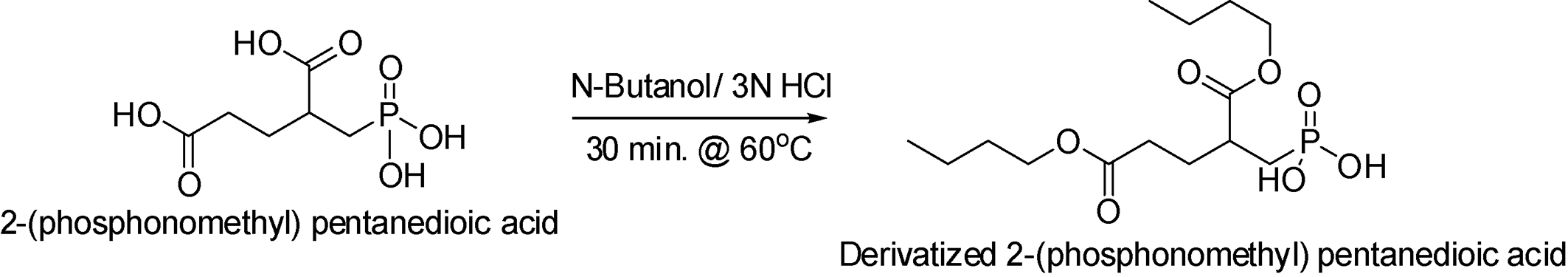

Supplement: S1 Fig — Reaction was carried out using n-butanol with 3N HCl at 60°C for 30 min, leading to formation of n-butyl esters of 2-PMPA carboxylic acids. (TIF) [file pone.0131861.s002.tif]
