# Supplementary material for: Selective CNS Uptake of the GCP-II Inhibitor 2-PMPA following Intranasal Administration
Source: PLoS One. 2015 Jul 7;10(7):e0131861. doi: 10.1371/journal.pone.0131861 (PMC4494705; doi:10.1371/journal.pone.0131861)
Supplement: S1 Protocol — Pharmacokinetics and behavioral characterization of proprictary small molecule therapeutics in rats. (PDF) [file pone.0131861.s003.pdf]

JOHNS HOPKINS
UNIVERSITY

Animal Care and Use Committee

1620 McElderry Street
Reed Hall, Room B122
Baltimore, Maryland 21205-1911
(443) 287-3738 / FAX (443) 287-3747
www.jhu.edu/animalcare

Dr. Barbara Slusher
Department of Neurology

Dear Dr. Slusher:

On 03/07/2013, the Johns Hopkins University Animal Care and Use Committee (ACUC) approved the following research protocol for which you are the Principal Investigator. A copy of the approved protocol is attached.

Protocol Number: RA13M65 Expiration Date: 03/07/2014

TITLE: Pharmacokinetics and behavioral characterization ofproprietary small molecule therapeutics in rats

The approval period is for one (1) year. The protocol may be renewed two times before complete review is needed. The ACUC office will send notices reminding you to submit either an annual renewal or a 3rd year replacement protocol. The notices will be sent out 90 days prior to the expiration date. Please use this protocol number when placing an order with Research Animal Resources (RAR) (formerly known as Animal Services). They can be contacted by calling 5-3713. Note: Approval of this protocol does not guarantee University space for housing animals.

You may request modifications to this protocol by submitting the appropriate amendment form (i.e., Change in Animal Number, Change in Personnel, or Change in Procedures) to the ACUC office for review and approval. Copies of all our forms can be found on our website www.jhu.edu/animalcare. For guidance on protocol modifications that require amendments, please refer to the reverse side of this letter. If the locations for outside housing or procedures change, please submit a Change in Location Form, also available on the website.

Sincerely,

Nancy A. Ator

Nancy A. Ator, PhD
Chair, Animal Care and Use Committee

Johns Hopkins University Animal Care and Use Committee (ACUC)

PROTOCOL FORM

Release Date: **7/9/2012**

For ACUC Use Only	
☐ Original	☑ Revision
PROTOCOL #:	RA13M65
DATE REC'D:	Aug. 1/23/13 · Rev. 3/1/13
EXPIRATION DATE:	3/7/14
☑ Logged	☑ Database
**BSL: 2 3**	**ABSL: 2 3**

New Protocol? x Yes ____ No

3rd Year Replacement of Protocol#: ____________

Do you currently have animals housed on this protocol? ____ Yes x No

PROTOCOL TITLE: Pharmacokinetic and Behavioral Characterization of Proprietary Small Molecule Therapeutics (eg GCPII inhibitors, DAAO inhibitors) in Rats

1. PRINCIPAL INVESTIGATOR (PI): Faculty member responsible for design and implementation of the research.

LAST NAME: Slusher **FIRST:** Barbara **M. INITIAL:** S **DEGREE (S):** PH.D

Title: Director Neurotranslational Program and Assoc Prof., Depts of Neurology and Psychiatry **School:** BSI/JHMI **Dep't/Div:** Neurotranslational Center and Dept of Neurology /Psychiatry

Campus Mail Address:

Building: Rangos **Room Number:** 277 **Campus :** East Baltimore

Phone: 410 614 0662 **Fax:** 410 614 0659 **E-mail:** bslusher@jhmi.edu

2. PRIMARY CO-INVESTIGATOR, if applicable: Person who is delegated authority when the PI is unavailable.

LAST NAME: Wozniak **FIRST:** Krystyna **M. INITIAL:** M **DEGREE (S):** Ph.D

Title: Med Translational Assoc **School:** BSI/JHMI **Dep't/Div:** Neurotranslational Center

Campus Mail Address:

Building: Rangos **Room Number:** 233 **Campus:** East Baltimore

Phone: 443 287 0178 **Fax:** 410 614 0659 **E-mail:** Kwoznia3@jhmi.edu

3. ADMINISTRATIVE CONTACT *(to whom the ACUC office may release protocol information if requested)*

Name: Angie Rubin **Phone:** 410 614 1065 **Fax:** 410 614 0659 **E-mail:** arubin12@jhmi.edu

4a. ANIMAL REQUIREMENTS: A separate protocol form is required for each species. List rodent strains separately. See questions 13 and 17a for giving numbers of animals that will be needed.

Genus/Species or Common Name	Strain and/or Stock	Sex	Source (e.g., commercial, another investigator, in-house breeding)	Indicate Age and/or Weight required
Rat	Wistar/Sprague Dawley	M and F	HSD	6-12 wks 200-250g

4b. Number of rats or mice that might be used from the euthanasia racks per year: O

These are cages of animals that have been marked for euthanasia that may be used for training, practice, or tissue harvest using procedures approved in this protocol. The use of the animals must be completed the same day they are taken. Your name will be added to a list of approved users, and you will be sent complete instructions if you are not already approved.

5. ANIMAL RESOURCES HOUSING: What is the range (lowest # to highest #) of the number of animals you predict to be housed in and cared for in central facilities at any given time? 0-50

The ACUC website, web.jhu.edu/animalcare, contains a formulary for rodent drug doses and ACUC-approved guidelines for many procedures. The guidelines may be cited as the method to be used instead of listing the information in this form if it is the same method to be used.

6. **SATELLITE HOUSING:** Will any animals be housed outside an Animal Resources Facility for more than 12 hours if the species is covered by the USDA regulations or for more than 24 hours if not? _____ **Yes** __X__ **No** If "Yes," complete the Satellite Housing Form (web.jhu.edu/animalcare) and submit as an attachment to this protocol.

7. **PROCEDURAL KEYWORD CHECKLIST:** *Please read each carefully and check all of the following that apply.* ***Provide requested details in the answer to question 14, unless another question number is indicated***. (Note: This list is not meant to be exhaustive. It is possible no items will be checked for this protocol.)

___ **Antibody production** – Method to be used (i.e., poly- or monoclonal). Tell whether ascites will be used.*
_X_ **Behavioral studies** – Methods and apparatus to be used for the purpose of explaining what the animals will experience.*
___ **Breeding colony** Note: If breeding mice, you will need the mouse breeding form off the website*
___ **Blood collection in survival procedures** – Site & methods of collection; quantity & frequency of collection.*
___ **Caging** – Special caging requirements (e.g., wire bottom, metabolic). Give reasons.
___ **Dietary manipulation** (i.e., testing the effects of manipulating the content of food or fluid)
___ **Environmental conditions** – That are non-standard (e.g., lack of light cycle, temperature &/or humidity outside of normal range)
___ **Euthanize and harvest tissue ONLY—No other procedures in this protocol.**
___ **Food or fluid restriction –** Give durations and information on monitoring weight/hydration status, as applicable*
___ **Imaging** – include frequency for individual animals if more than once
___ **Infectious disease** See question 19.
___ **Irradiation** – Describe expected effects on animals after procedure & duration. Describe precautions to protect personnel and animals in question 19.
___ **Human embryonic stem cells?** Must also submit application to JHU ESCRO.
_x_ **Non-survival surgery**
___ **Paralyzing agents** – must be used in conjunction with anesthesia; include information on how depth will be monitored.
___ **Restraint (other than by hand or briefly)** – Method, rationale, procedures for habituation, durations, & monitoring.*
___ **Recombinant DNA and/or biohazard.** See question 19
___ **Stress** (as the topic of study; e.g., via cold exposure, restraint, forced exercise, uncontrollable aversive stimuli) Explain rationale & why this particular method is being used.
___ **Survival surgery** See question 15*
___ **Teaching** – Type of class(es) or students
_x_ **Toxicology or study of effects of chronic drug**– Classes or specific substances to be used; give route(s), sites, & frequencies of administration. Give expected untoward effects & their duration and if known.
___ **Tumors** experimentally induced or transplanted – Expected maximum size & plan for monitoring. If xenografts, identify source.*

***See guideline at:** web.jhu.edu/animalcare

8. **LOCATIONS OF PROCEDURES TO BE PERFORMED ON LIVE ANIMALS:** If procedures will be performed in an Animal Resources facility, indicate "AR" in place of the room number.

Procedures performed on live animals:	Building(s)	Room Number(s)	Campus
Survival surgery (Major & Minor)	Meyer	234	East Baltimore
Non-survival surgery or Euthanize & harvest	Meyer	234	East Baltimore
Behavioral testing	Meyer	234	East Baltimore
Imaging			
Other (Dosing)		AR	

In answering the questions below, please use terminology that will be understood by a non-specialist. Spell out abbreviations on first use.

9. **OBJECTIVE(S):** Briefly explain the overall purpose of the project.

The small molecule therapeutic (novel/proprietary drug) is to be administered to rats by various routes that would be applicable to use in humans, and the disposition, pharmacodynamic and/or pharmacokinetic activity of the compounds are to be determined. This information is crucial for determining the viability of a compound for preclinical and ultimate clinical testing (e.g. a compound that is not absorbed or does not reach the target tissue in appreciable amounts would not be developed further, despite possible activity in vitro). In addition, initial behavioral

characterization including gross whole animal activities associated with the test compound would be determined by non-invasive methods. Gross effects on behavior would likely prevent further characterization.

Two major classes of drugs that may be tested include: Inhibitors of Glutamate Carboxypeptidase and D-Amino Acid Oxidase. Inhibition of these enzymes reduces glutamate (involved in pain) and increases D-serine levels (involved in schizophrenia) respectively.

10. **IMPORTANCE OF RESEARCH:** What is the relevance of this work for human or animal health, the advancement of knowledge, or the good of society?

This work will give us important information on the penetration of the drug and its bioavailablility so we can rule out non-suitable candidates (with poor penetration) early in development or promote interesting entities into further testing and development. Discovery of a novel therapeutic could ultimately benefit both duration human and animal disease sufferers, alike.

11. **RATIONALE FOR ANIMAL USE:** Why are live animals necessary for this study? *Include the reason a non-animal approach such as mathematical models or computer simulation could not be used.*

The amount of drug absorbed by the organism requires a living organism to quantify, as it is subject to absorption and metabolism by various organs. A non bioavailable compound is useless as a biological entity as the whole organism is what will be required to treat and these can be identified discarded from further discovery using these studies.

12. **SPECIES SELECTION:** Explain why you selected this species as opposed to another.

Rodents are small and relatively inexpensive. Rats are slightly larger than mice and are needed for certain routes of administration eg., intranasal, since the nasal cavity is too small in mice. Also if a larger blood volume is needed for analysis, this can be more readily obtained from a rat over a mouse.

13. **NUMBERS OF ANIMALS AND RATIONALE:** **<u>Explain how many animals (or range of animals) are needed for each experimental condition. State how you determined that this number is appropriate</u>**. The answer should be in relation to the experimental design and statistical significance, if applicable to the design. Use a table showing experimental and control groups if it aids communication. If the total number of animals will not match up with the total for question 17a, please explain.

The total number of animals for PK and tissue distribution studies is based on an n of 15 per compound with 3 rats per time point for 5 pre-determined time points. Our experience has found this gives us sufficient numbers to initially evaluate the effects of the drugs we are testing. Total compounds per year estimated at 15 giving total of 225 per year for 3 years. Of these we estimate about 30 rats per year will be subjected to intranasal drug administration

Another 4 experiments utilizing 20 rats per study or 80 rats total (n=5 per group) are anticipated per year for behavioral assessments with acute or chronic (5 day) dosing.

14a. **DESCRIBE THE PROPOSED PROCEDURES:** Provide a description of procedures to be carried out on living animals. **The answer needs to make clear when more than one procedure will be carried out on the same animal. Refer to prompts in question 7 for details of specific procedures to include. <u>State which survival surgeries will be performed, but save details of each survival surgery and post-operative care for question 15.</u> <u>Save details of euthanasia for question 16d.</u> Do include details of non-survival surgery here.** ***Further Guidance:*** ***a)*** *For* ***blood sampling****, include possible sites, quantities and frequencies of collection.* ***b)*** *For* ***pharmacological assessments*** *that will involve multiple compounds when the names of the compounds are not yet known, identify the groups by chemical class or mechanism of action. Provide the basis on which dose ranges will be determined if specific doses are not yet known. Indicate planned or possible routes of administration.* ***c)*** *Give ranges of durations of experimental conditions and other parameters unless they are not likely to change or fluctuate.* ***d)*** *Think ahead to minimize the need to submit amendments later if the possibility of other procedures is likely.*

The ACUC website <u>web.jhu.edu/animalcare</u> contains a formulary for rodent drug dose and ACUC-approved guidelines for many procedures. If you are following these, you can cite them as the method to be used instead of listing all the information in the form.

3 Potential Experimental Paths are proposed:

A-Behavioral Determination or, B- Pharmacokinetic or C-Tissue Distribution Characterization.

For each compound, the proprietary test articles will be administered by one or more of the following routes:

Routes of Administration

Oral- For oral administration animals are to be dosed using a curved gavage needle (20 gauge) for consistency in deposition of the test article. Animals typically will receive a dose of 1–100mg/kg of test article but could be more or less depending on experimental needs. Dose volume is to be 10 ml/kg. The vehicle used for the test article may contain PBS, saline, PEG / ethanol / water or other vehicle, or any combination of these, to ensure the compound stays in solution.

IV- Intravenous administration is to be conducted by injection into one of the lateral tail veins. Animals will be warmed by IR light to promote the appearance and exteriorization of the caudal vein on the surface of the tail. The rat will be placed into an approved restrainer during the injection. This helps to prevent the rat from jerking the needle out of the vein during the injection, minimizing risk of injury to the rat and the technician. A 22g needle will then be inserted into the vein as distally as possible and the injection made into the vein. Dose volume typically is to be1ml/kg. The animal is then promptly removed from the restraint and returned to its home cage.

IP and SC.- The majority of experiments are to be conducted using oral or I.V. administration but it may be necessary to administer a test article via intraperitoneal (IP), subcutaneous (SC) or intramuscular (IM) routes, using the smallest gauge needle possible to deliver the test article. This helps to minimize discomfort to the animal and prevent backflow of dosing solution. The region between the shoulder blades is typically the site of injection for SC administration. The lower right quadrant is the preferred location for IP injections. Animals may receive up to 100 mgs or more of test article per kilogram of body weight in no more than 10 ml/kg of dosing solution.

Intranasal (IN)- In certain cases, we will need to administer drugs via the intranasal route (single administration only).

For initial experiments to show availability via the intranasal route we will need to conduct surgery as described below and widely published on (see Drug Development and Delivery Vol. 4, January/February 2004; Journal of Pharmaceutical Sciences, Vol. 90, 1729-1735 2001; Acta Pharmacol Sin, Vol 25, 522-527, 2004; Cancer Chemother Pharmacol , 2006, 57: 97–104). Rats will be anesthetized with chloral hydrate given IP at an initial dose of 1 ml of a 10% solution. Supplementation of 0.1-0.2 ml will be provided as necessary. In our experience choral hydrate serves our needs best for a non-inhalation anesthetic that can be used to keep an animal anesthetised for up to 6 h. Lack of corneal reflexes or response to tail pinch will be used to ensure adequate depth of anesthesia for the duration of the procedure. Respiration and color of mucus membranes will be assessed throughout the procedure to determine airway patency.

An incision will be made along the neck, the trachea isolated and transected. The upper part will be tied off with a 3-0 silk suture, and the lower part cannulated with PE240 tubing to aid air breathing. The esophagus will be isolated and tied off with a 3-0 silk suture. Rats will be maintained lying on their back, and in this position, the animals will be given 10ul of solution per nostril using a micro-syringe connected to 1.5cm PE-10 tube of the experimental drug solution. This surgery is needed to prevent drainage of nasally applied dosing solution to non-nasal regions. Throughout the entire experiment, all rats will remain lying on the back, and in this position, drug solutions can contact a larger area of nasal olfactory mucosa compared to the prone position.

At the end of the experiment, the animals will be euthanized with CO2, and blood will removed by cardiac puncture and brain collected. For experiments involving intranasal administration, experiments will be terminated after a maximum of 6 hrs, since anesthesia must be maintained for the duration of the experiment, in view of compromised air passages i.e., this is non-survival.

After results from initial studies confirm availability of a test compound via the intranasal route, subsequent studies may be performed using a more "crude" preparation with a rat acutely anesthetized (with isoflurane) and direct administration of the compound performed intranasally (without surgery).

Topical- In certain cases, we would like to potentially apply proprietary compounds in a liquid topical administration to the hind paw. These would be compounds potentially to be developed as analgesics as analgesic creams are often used. For these studies we propose anesthetizing the rats with isoflurane and dipping their hind paws in a non-

irritant solution of drug for 5, 15 or 30 mins, with anesthesia maintained for the duration of exposure. To evaluate the passage of the compound into the skin we will conduct studies in rats after acute or 5 days of compound administration in this manner. Thereafter (within 24 h of last drug exposure) the rats will be euthanized and skin punches (3-5mm) removed from the hind paws for analysis of compound levels.

POSSIBLE EXPERIMENTAL PATHS:

EXPERIMENTAL PATH A: BEHAVIORAL ASSESSMENT
Rats will be dosed as described above (using oral and/or ip routes only) and evaluated for general behavioral effects of the proprietary small molecule by a non-invasive procedure including possible assessment of general locomotion, motor co-ordination, righting reflex etc. These assessments will be made by general observation of behavior and not by use of any testing apparatus.

Animals will be observed at various time points after the test article administration, up to 6 hours after a single acute administration or in certain cases, 5 daily doses of the test compound. Multiple dose studies will only be performed with oral, ip or iv administered compounds. Any animal showing signs of undue stress or discomfort such as vocalization, gasping, inability to move etc will be euthanized immediately and similarly treated animals closely monitored.

EXPERIMENTAL PATH B: PHARMACOKINETIC ASSESSMENT
Animals are to be dosed as described above and terminal blood collected at selected time points based on test article characteristics and experimental needs. Pre-dose (PD) or "control" samples are collected from naive animals. Screening experiments may only require N=2-3 per time point to verify the presence of a test article in plasma at specified time points. Below is a typical time course for blood collection but not all time points will be included in each study. We anticipate 6 of these multiple time points being predetermined for use per study.
P.O. experiments: PD, 15, 30, 60, 90, 120, 240, 360, 480 min., and 24 hours.
I.V. experiments: PD, 5, 15, 30, 60, 120, 240, 360, 480 min., and 24 hours.
IM, IN, IP, and SC. experiments: PD, 15, 30, 60, 90, 120, 240, 360, 480 min.
Topical: PD, 15, 30, 60, 90, 120 min.

Sampling-Blood Collection
Terminal bleeds are to be performed via cardiac stick (using a 25g or 26g needle attached to a 1-ml syringe) following euthanasia by CO2 inhalation. Approximately 0.5 – 1.0 ml of blood will be collected from each animal.

EXPERIMENTAL PATH C-TISSUE DISTRIBUTION
Animals will be dosed as previously described and tissues harvested based on test article characteristics and experimental needs. Some experiments may be limited to harvesting a single organ, such as the brain, while other experiments may require the collection of several organs. Tissues that might normally be selected for harvesting and subsequent analysis include (but are not limited to): brain, heart, liver, kidneys, and sciatic nerve. Pre-dose (PD) or "control" samples are collected from naive animals.

Although the number of animals used per experiment may vary depending on experimental needs, the number of animals used is to be kept to a minimum. For example, Screening experiments may only require N=1 per time point, just to verify the presence of a test article in a target organ, whereas an experiment to determine detailed pharmacokinetic concentrations of a selected compound in a target organ(s) or tissue may require the use of more animals per time point (typically N=3).

Sample collection and handling
At each sampling time point animals are to be euthanized via CO2 inhalation and blood collected via cardiac puncture. Selected tissues are then to be harvested, weighed, and placed in a vial and flash frozen or placed in a vial containing 3x the tissue weight of phosphate buffered saline. These vials will then be stored at–80o C until analysis.

14b. WILL ALL DRUGS USED FOR SEDATION, ANESTHESIA, OR ANALGESIA IN CONJUNCTION WITH ANY PROCEDURES NAMED IN THIS PROTOCOL (#14a, 15, 16d) BE "PHARMACEUTICAL GRADE"?
___X__ **YES** ______ **NO** Formulations used for these purposes must be those sold for clinical use (i.e., "pharmaceutical grade") unless an alternative formulation (i.e., "non-pharmaceutical grade") is necessary due to (1) scientific considerations or (2) non-availability of the preferred compound in a clinical use formulation. If the answer above is "NO," **state below the name of any drugs for these purposes that may not be pharmaceutical grade and your reason for using the non-pharmaceutical grade version.** ****See "Non-pharmaceutical Grade Drug Policy" at web.jhu.edu/animalcare for full definitions of "pharmaceutical grade" and exceptions and for JHU requirements on preparation and storage.****

When needed (eg for intranasal administration) the anesthesia drugs (except for chloral hydrate) will be pharmaceutical grade. Isoflurane cannot be used for IN due to the potential long duration of anesthesia and for intranasal administration rats will be tracheotomized. We request use of chloral hydrate as this has a less steep dose-response than ketamine/xylazine and so in our experience, is easier to administer for longer anesthesia (over several hours).

14c. DRUGS/CHEMICALS USED AS RESEARCH TOOLS OR THE SUBJECT OF INVESTIGATION. If formulations will not be "pharmaceutical grade," state below the name/class of the compound and the reason that non-pharmaceutical grade is necessary. **Reasons could include: (1) non-availability in pharmaceutical grade, (2) scientific considerations (e.g., lack of suitable vehicle control, presence of preservatives, necessity of manipulating concentration, to be comparable with previous studies), (3) use of drugs provided by NIH drug supply program.** ****See "Non-pharmaceutical Grade Drug Policy" at web.jhu.edu/animalcare for full policy and requirements for preparation and storage.****

All test agents, since proprietary and made by in-house chemists, will be non-pharmaceutical grade as no other grade is available.

14d. MULTIPLE SURVIVAL SURGERIES: If you indicated in 14a that a survival surgery (i.e., any procedure involving an incision) will occur, state whether any animal will undergo more than one survival surgery (i.e., involving separate periods of anesthesia and recovery)? This includes any surgeries yet to be fully described in #15.
______ **Yes** ___X___ **No** If "Yes", the order and the interval (or range of intervals) between them should be included in 14a. Below, provide the scientific justification for multiple survival surgeries in the same animal. (Note: Neither cost savings alone nor reduction of the number of animals needed is considered adequate.)

14e. BREEDING: Will **mice** be bred at JHU under this protocol? _______ **Yes** ______X_ **No** (If **"Yes"**, complete **Mouse Breeding Colony Form** (web.jhu.edu/animalcare). **If species other than mice will be bred under this protocol, please provide details (i.e., projected number of offspring per year; breeding age range; weaning age; genotyping, if applicable; special care; etc.) below:**

15. SURVIVAL SURGERY: *(If more than one type will be performed, fill out a separate question 15 for each one.)*
Name of Surgical Procedure: _**None** ________________________________

15a. MAJOR OPERATIVE PROCEDURE? Will this surgery penetrate and expose a major body cavity or cause substantial impairment of physical or physiologic function? _____ **Yes** ___X_ **No**

15b. Pre-anesthetic agents (e.g., to permit handling, intubation). ***Name, dose, and route.***
None

15c. Pre-emptive analgesia (i.e., analgesia given prior to the surgical procedure). ***Name, dose, and route.***

15d. Anesthesia. ***Name, dose, and route.*** **Also state the method that will be used to assure the animal is anesthetized prior to incision.**

The ACUC website web.jhu.edu/animalcare contains a formulary for rodent drug dose and ACUC-approved guidelines for many procedures. If you are following these, you can cite them as the method to be used instead of listing all the information in the form.

15e. Will a neuromuscular blocking agent (paralytic) (i.e., one that prevents respiration) be used at any point in the procedure? _____ Yes ___x__ No If "Yes", state name, dose, route and frequency of administration of paralytic agent(s). **What parameter(s) will be used to determine that the animal remains anesthetized after the paralytic has been administered?**

15f. Describe intra-operative procedures including: monitoring, surgical procedure, method of wound closure, etc. Must be aseptic technique including assurance regarding the use of gown, gloves, mask, and sterilized instruments. Include name, dose, and route of any intra-operative analgesia.

15g. Describe immediate post-operative care (first 24 hours). Include monitoring, supportive care, and analgesia.

15h. Describe post-operative care procedures after the first 24 hours, including plan for monitoring, suture removal, further analgesia, special feeding, etc:

16a. PLANNED ENDPOINT/EUTHANASIA: If not already described in question 14, state the timepoint or other criterion in the experiment at which euthanasia will occur for each animal or experimental group if the study goes as planned.

Animals will be euthanized immediately prior to blood/tissue withdrawal, as described in Section 14a. In most cases this will be within 8 h of receiving a single dose of compound.

If used in behavioral studies they will be euthanized immediately after observation period has been completed. For the 5 -7 day subchronic behavioral studies, rats will be euthanized within 24 h after the last test dose,

Bilateral thoracotomy will be performed as a secondary euthanasia method following CO2 asphyxiation after blood withdrawal.

16b. If euthanasia is not required by the study, or for particular animals, indicate the possible disposition of the animals (e.g., adoption, transfer to another study).

All animals will be euthanized on experimental completion.

16c. Give the health conditions and/or criteria under which early euthanasia (withdrawal of an animal from the study) will be considered.

In the multiple dose studies described, rats will be weighed daily. If in any of these 5 day studies we determine that >20% individual weight loss has occurred or that animals show clinical signs of weakness/distress animals will be euthanized immediately.

16d. What method(s) of euthanasia will be used by laboratory personnel? Also state how death will be verified before disposal (give two criteria if euthanasia is other than decapitation). *See JHU's Euthanasia Guideline at web.jhu.edu/animalcare for suggested methods.*

Animals will be euthanized by a lethal overdose of CO_2 followed by bilateral thoractomy

17a. PAIN/DISTRESS: Indicate in the table below, the approximate numbers of animals that will fall in each Pain/Distress category. In *addressing pain and distress, please consider all aspects of the study (e.g., surgery, phenotype of the animal, induced disease or tumor burden, behavioral procedures).* For protocols that involve more than one procedure, place animals in the category that pertains to the greatest degree of pain, distress and/or discomfort to which the animal will be exposed. Do not count an animal more than once. The total number of animals entered must agree with the total animals in question 13 unless the discrepancy is explained there. The total should not include the number in answer to question 4b. Use of anesthesia for rodent tail snips or purely for restraint (e.g., for imaging) does not require placement in Category D.

The ACUC website web.jhu.edu/animalcare contains a formulary for rodent drug dose and ACUC-approved guidelines for many procedures. If you are following these, you can cite them as the method to be used instead of listing all the information in the form.

Categories	Examples	Total Number of animals for 3 years (Entered into ACUC database at time of protocol approval.)
B—Breeding	e.g., number of males and females to be used for breeding	
C—Procedures cause momentary, slight, or no pain/distress in absence of analgesia or anesthesia	e.g., injections, euthanasia, blood collection, brief restraint, imaging	825
D—Procedures potentially are painful but anesthetics and/or analgesics are given	e.g., surgery, blood collection by invasive routes	90
E—Procedures involve pain/distress that will not be alleviated by drugs.	e.g., toxicity studies, pain or stress studies, some disease models	

****** Answer 17b and c below only if any animals fall into Category D or E.******

17b. What procedure(s) or other elements in this protocol fit the definition of Category D and/or E as given in the chart above?

Intranasal administration route for test article administration.

17c. Are there are alternative methods to those you named in 17b that you could use that would produce less pain and distress and achieve the same experimental purpose? Either state approaches that might seem to be reasonable alternatives to the to the ones in 17b, and explain why they will not accomplish the experimental objective with less pain/distress or carry out a Keyword/Literature Search. If you choose the first approach, please then state the reasons you can be confident that you have relevant and up-to-date information on the topic. Reasons include: 1) consultation with an expert in the research area (give name/qualifications), 2) regular attendance at scientific meetings (names of organizations), 3) regular attention to the scientific literature on the topic (cite sample journal names), and/or 4) personal experience with the alternative method.

We have consulted with some experts in the area of intranasal delivery and there are several publications cited in Section 14a, describing the surgical methods described for intranasal delivery, required to prevent nonspecific spill-over of intranasally administered compound into trachea and oesophagus. There is no other method available that would produce less pain and distress. This is a non-survival surgery.

If you do a Keyword/Literature search, provide the following information:

Date (day, month, year) search was performed: ______________

Years covered by search: ___________________

Keywords used in search: These must be in relation to the procedure(s) named in 17b.

Databases searched (check all that apply):
____ MEDLINE ____ AWIC ____ TOXLINE ___ AGRICOLA ____ Other (describe)

Did the literature search reveal one or more alternatives that cause less pain/distress than the methods proposed but accomplish the scientific purpose? ____ Yes ___ No If yes, explain why the alternative is not being used.

18. ENVIRONMENTAL ENRICHMENT: Requests for Exemption from the JHU program. *See website link in footnote for detailed information on the JHU Environmental Enrichment plan for all species. Unless an exemption is approved, the animals under this protocol will be included in the campus-wide plan for this species.*

ALL SPECIES:

The ACUC website web.jhu.edu/animalcare contains a formulary for rodent drug dose and ACUC-approved guidelines for many procedures. If you are following these, you can cite them as the method to be used instead of listing all the information in the form.

a. Most animals will be provided toys and/or other non-food enrichment items (such as nesting material for mice). Are you seeking an exemption for scientific reasons for any portion of the work covered in this protocol?
______**Yes** __x___ **No** If "Yes," explain below.

b. Most animals (other than mice and rats) are periodically provided edible enrichment. Are you seeking an exemption for scientific reasons for any portion of the work covered in this protocol?
_____**Yes** ___x__**No** ______ **Not Applicable.** If "Yes," explain below.

c. Will you be providing edible or other enrichment beyond or in place of that provided by central facilities for your animals? ______ **Yes** __x___ **No** If "Yes", state how.

d. Most animals will be housed routinely in pairs or groups with others of their species. Are you seeking an exemption for scientific reasons from JHU's social housing plan for this species for any portion of the work covered in this protocol? ______ **Yes** _x____ **No** If "Yes" explain below. **Note**: The Attending Veterinarian may exempt your animals from social housing for health or other well-being concerns even if you do not seek an exemption for scientific reasons.

DOGS:

e. If this protocol covers dogs, are you seeking an exemption for scientific reasons from JHU's exercise program?
______ **Yes** ______ **No** _____x_ **Not Applicable**. If "Yes," state reason.

19. **RECOMBINANT, INFECTIOUS AND/OR BIOHAZARDOUS AGENTS USED IN ANIMALS.** Use of certain agents requires approval from Health, Safety & Environment's Institutional Biosafety Office/Committee (410-955-5918). If Biosafety Committee approval is pending, attach a copy of the IBC application.

Type of Hazardous Agent	Yes	No	Name of Agent	Current Approval Date	Biosafety Regis. # or Radiation Safety #
Recombinant, plasmid or "naked" DNA					
Virus or viral vectors					
Bacteria, parasites or other pathogens					
Human tissues or cell lines					
Radioactive compounds					
Hazardous chemicals or toxins (e.g., lead)				Not applicable	Not applicable

***NOTE*: If the Institutional Biosafety Committee (IBC) determines that an animal given the agent(s) named above has the potential to be hazardous to people (e.g., through exposure to its urine or feces), you are required to label the cage with a biohazard card at the time of initial exposure and for the duration of hazardous effects as determined by the IBC. Instructions will be included with the Approval Letter for this protocol.**

20. **TRAINING & QUALIFICATIONS: Provide specific information on training and/or experience that qualifies each person to perform the procedures involving the species of animal in this protocol.** Make additional copies of these pages as necessary.

If any individuals listed below have not previously been included on an approved protocol at JHU, they must complete the online Animal Care and Use training course (found at https://secure.lwservers.net/default.cfm) and enroll in the Animal Exposure Surveillance Program (AESP) before the protocol can be approved (contact

Occupational Health at 410-955-6211). *Copies of the Online Training Certificate and the AESP certificate of enrollment for these individuals must be supplied to the ACUC Office before final approval of the protocol may be granted.*

Note: For protocols used only for Teaching/Training, trainees do not need to be listed in question 20.

Role: **Principal Investigator**

Last Name: Slusher First Name: Barbara M. Initial:S

1- Degree(s): Ph.D

2- Which procedures will this person be performing (can state "all")?
None

3- How much experience with these procedures generally and in this species does the person have?
Completion of all JHU animal training courses

ACUC office use: AESP ✓ **OLT** ✓

Role: **Primary Co-investigator (person delegated authority when PI is unavailable)**

Last Name: Wozniak First Name: Krystyna M. Initial:M

1- Degree(s): Ph.D

2- Which procedures will this person be performing (can state "all")? All except for surgery for IN studies

3- How much experience with these procedures generally and in this species does the person have?

Extensive experience in animal handling and experimentation (30 years) and completion of all JHU animal care and use courses. No training supervision required for any other procedures except for intranasal surgery as Krystyna has prior experience with them.Krystyna will not conduct surgery for intranasal administrations.

ACUC office use: AESP ✓ **OLT** ✓

Role: X☐ **Co-Investigator** ☐ **Fellow** ☐ **Student** ☐ **Faculty** X☐ **Staff** ☐ **Outside Collaborator** (Check all that apply.)

Last Name: Wu First Name: Ying M. Initial:

Department: Neurotranslational program BSI/JHMI Phone Number: 410 614 7280

JHU Address: 855 N Wolfe Street, Baltimore, MD Bldg & Room: Rangos 265

Email Address: Ywu58@jhmi.edu

1- Degree(s): BS

2- Which procedures will this person be performing (can state "all")?ALL

3- How much experience with these procedures generally and in this species does the person have? If training and/or supervision of this person is needed, who will be providing it?
20 years experience at JHU and Pharma in animal experimental techniques. No training or supervision required. Ying has prior experience with the intranasal surgery as instructed by Maya George who is working in drug delivery at University of Iowa.

ACUC office use: AESP ✓ OLT ✓

Role: x☐ Co-Investigator x ☐ Fellow ☐ Student ☐ Faculty ☐ Staff ☐ Outside Collaborator (Check all that apply.)

Last Name: Potter First Name: Michelle M. Initial:

Department: Neurology Phone Number: 410 614 1005

JHU Address: 855 N Wolfe Street, Baltimore, MD Bldg & Room: Rangos 227

Email Address: Mpotte10@jhmi.edu

1- Degree(s): Ph.D

2- Which procedures will this person be performing (can state "all")? All except for surgery for IN studies.

3- How much experience with these procedures generally and in this species does the person have? If training and/or supervision of this person is needed, who will be providing it?

Completed all JHU animal training courses. No training or supervision required for other procedures as direct experience with all these in past.

ACUC office use: AESP ✓ OLT ✓

Role: ☐ Co-Investigator ☐ Fellow ☐ Student ☐ Faculty ☐ Staff ☐ Outside Collaborator (Check all that apply.)

Last Name: First Name: M. Initial:

Department: Phone Number:

JHU Address: Bldg & Room:

Email Address:

1- Degree(s):

2- Which procedures will this person be performing (can state "all")?

3- How much experience with these procedures generally and in this species does the person have? If training and/or supervision of this person is needed, who will be providing it?

ACUC office use: AESP __ OLT __

Role: ☐ Co-Investigator ☐ Fellow ☐ Student ☐ Faculty ☐ Staff ☐ Outside Collaborator (Check all that apply.)

Last Name: First Name: M. Initial:

Department: Phone Number:

JHU Address: Bldg & Room:

Email Address:

1- Degree(s):

2- Which procedures will this person be performing (can state "all")?

3- How much experience with these procedures generally and in this species does the person have? If training and/or supervision of this person is needed, who will be providing it?

The ACUC website web.jhu.edu/animalcare contains a formulary for rodent drug dose and ACUC-approved guidelines for many procedures. If you are following these, you can cite them as the method to be used instead of listing all the information in the form.

Principal Investigator's Signature: [signature] **Date:** 1/22/13

22. APPROVAL:

IACUC Chair's Signature: Nancy A. Ator **Date:** 3/7/13

Please submit to the JHU ACUC office (please do not staple):
Reed Hall B122, East Baltimore Campus.
If you prefer to fax, please use only the last five numbers if dialing within Hopkins (7-3747).
Use the entire number only if faxing from outside of Hopkins (443-287-3747).

If you fax the protocol and choose also to send the original, please put a cover sheet on the original indicating that the protocol previously was faxed so that it is not logged in as a new submission. Thank you.

The ACUC website web.jhu.edu/animalcare contains a formulary for rodent drug dose and ACUC-approved guidelines for many procedures. If you are following these, you can cite them as the method to be used instead of listing all the information in the form.
